# The Influence of Temperature on Anisotropic Wettability Revealed by Friction Force Measurement

**DOI:** 10.3390/biomimetics8020180

**Published:** 2023-04-25

**Authors:** Zhen Lin, Kangjian Xiao, Lijun Li, Yurong Zhang, Xiaolong Zhang, Daobing Chen, Longjian Xue

**Affiliations:** 1 School of Power and Mechanical Engineering, Wuhan University, South Donghu Road 8, Wuhan 430072, China; 2 The Institute of Technological Science, Wuhan University, South Donghu Road 8, Wuhan 430072, China

**Keywords:** anisotropic surface, liquid-solid friction, graphene, wettability, bioinspired

## Abstract

Anisotropic surfaces with special wettability under various temperatures are of both fundamental interest and practical importance in many fields. However, little attention has been paid to the surfaces at temperatures between room temperature and the boiling point of water, which is partially due to the lack of a suitable characterization technique. Here, using the MPCP (monitoring of the position of the capillary’s projection) technique, the influence of the temperature on the friction of a water droplet on the graphene-PDMS (GP) micropillar array (GP-MA) is investigated. The friction forces in the orthogonal directions and the anisotropy in the friction decrease when the GP-MA surface is heated up, based on the photothermal effect of graphene. The friction forces also decrease along the pre-stretching direction but increase in the orthogonal direction when the stretching is increased. The change in the contact area, the Marangoni flow inside a droplet, and the mass reduction are responsible for the temperature dependence. The findings strengthen our fundamental understanding of the dynamics of drop friction at high temperatures and could pave the way for the design of new functional surfaces with special wettabilities.

## 1. Introduction

Anisotropic wetting originating from anisotropic surface structure or arrangement provides vital functions for many plants and animals to survive in nature [1,2,3,4,5]. For instance, with the asymmetric hierarchical topography of the peristome surface, the pitcher plant is able to achieve directional liquid transport to accumulate nectar and water and even to form a slippery liquid film for trapping insects [6]. Anisotropic wettability also enables fish to reduce drag [7], water striders to walk on water [8], and beetles to capture water in the desert [9]. In addition, the wettability can change once the temperature of the surface or the environment changes, which has attracted increasing attention in recent years [10,11,12]. For instance, with the one-dimensional distribution of the mastoid structure in the parallel direction of leaf veins and uneven distribution in the vertical direction [3], water droplets accumulate on rice leaf when the temperature is low in the morning and roll along the veins as the temperature increases. The study of the influence of the temperature on the anisotropy surface wettability is thus of great importance in fundamental research and engineering fields, such as water accumulation, spray cooling, droplet transport, drag reduction, and agricultural spray [13,14,15,16,17].

Under various temperatures, the surface anisotropy can be easily regulated. At room temperature, the anisotropic adhesion of a water droplet on surfaces composed of an array of triangular pillars or stripes can be regulated in situ by the mechanical stretching of the elastic substrate [18,19,20,21]. Making use of the shape memory effect of the materials, smart surfaces with reversible isotropic/anisotropic wettability achieved the control of droplet motion by mechanical force or other stimulations [22,23,24]. With the photothermal effect, the temperature of a water droplet can be regulated locally, which breaks up the wetting symmetry of the droplet and thus manipulates the droplet motion [25,26,27,28,29]. Adding polypyrrole nanoparticles into a water droplet, Wooh et al. [25] were able to drive the droplet on a lubricant-impregnated surface and superamphiphobic surface with the focused irradiation of near-infrared light on the droplet. Once the temperature of the substrate could be heated up by light irradiation, the droplet temperature and therefore the droplet motion could be finely regulated. When the infrared-light irradiation was focused on the substrate at one side of a droplet, a temperature difference was generated between the two sides of the droplet, which resulted in the unbalanced surface tension and Marangoni force, driving the droplet towards the side without light irradiation [26]. When the surface temperature was close to the Leidenfrost point, the droplet levitated on a vapor layer; the movements of a droplet on surfaces have been intensively investigated [10,11,13,30]. The existence of a vapor layer would result in a negligible normal adhesion or lateral friction of the water droplet on the surface, which, however, would cause a high thermal resistance. Wang et al. [13] fabricated a micropillar surface with gradient periods and thus the coefficient of heat transfer and realized the directional transport of a high-temperature (close to Leidenfrost point) droplet towards the region with a higher heat-transfer coefficient. Liu et al. [11] found an interesting phenomenon in which a droplet showed a steerable bouncing on heated concentric microgrooves arrays under different temperatures, which is believed to originate from the synergistic action of the surface structure and boiling states. That is, the motion of a water droplet could be manipulated by controlling the temperature (close to the Leidenfrost point) and the topography substrate surface. On the other hand, the motion of a droplet on asymmetric surfaces with a temperature higher than room temperature but lower than the boiling temperature has required more investigation, though the phenomenon is quite common in our daily life. We assume the lack of a suitable technique to characterize the droplet motion within such a temperature window may partially be responsible for this situation.

Here, we investigate the influence of the temperature on the friction of a water droplet on a graphene-PDMS (GP) micropillar array (GP-MA) by MPCP (monitoring of the position of the capillary’s projection) technique [20]. The temperature of the GP-MA is regulated by the photothermal effect of graphene in the micropillars. While the water contact angle (CA) and sliding angle (SA) show a negligible dependence on the temperature (between room temperature and boiling point of water) or the surface geometries, the friction measurements reveal a clear change when the period and temperature of the GP-MA are changed. As the temperature increases, the friction force and the anisotropy along the orthogonal directions decrease. The results offer us the chance to better explore the surfaces with anisotropic liquid-solid friction.

## 2. Materials and Methods

### 2.1. Materials and Preparation

The PDMS elastomer kit (Sylgard 184) was purchased from Dow Corning (Midland, MI, USA). Graphene sheets were purchased from Aladdin (Shanghai, China).

The fabrication of the GP-MA samples contained the preparation of GP micropillars (demolded from PDMS mold) and pre-stretched PDMS film. A PDMS mold containing an array of micro-holes with 7 × 7 mm^2^ area, 50 μm in diameter, 20 μm in depth, and 70 μm in period was acquired via a conventional soft lithography technique. The PDMS precursor was prepared by mixing the base prepolymer and the cross linker in a weight ratio of 10:1. Graphene was added to the as-prepared PDMS precursor at a concentration of 0.8 wt%, according to our previous study, to form the GP precursor [31]. After stirring for 30 min, the GP precursor was degassed in a desiccator for 10 min and was filled into the PDMS mold to prepare the GP micropillars. The PDMS film was prepared by filling the as-prepared PDMS precursor into a glass chamber template, followed by curing at 90 °C for 1 h. After peeling from the template, the fully cured PDMS film with a thickness of 0.5 mm was cut into a rectangular shape (30 × 10 mm) and mechanically stretched to the predefined degrees. The pre-stretched PDMS film tightly covered the GP precursor-filled mold, followed by a curing at 90 °C for 1 h. After the demolding and relaxation of stress, the GP-MA sample was successfully fabricated.

### 2.2. Characterization

The morphologies of the graphene were examined by a field emission scanning electron microscope (MIRA 3 LMH, Tescan AG, Brno, Czech Republic) and an atomic force microscope (AFM, Nano Wizard 4, JPK Inc., Germany) in tapping mode (QI mode, scan rate = 5 Hz). The Raman spectroscopy was carried out by a laser micro-Raman spectrometer (Renishaw, English, Sheffield, UK) with an excitation wavelength of 532 nm.

The microstructures of the GP-MA were observed by dark-field optical microscopy (ECLIPSE Ci-L, Tokyo, Japan). The 3D structure of the GP-MA and the roughness of the micropillar top were characterized by a white light interference 3D profiler (New View TM 9000, ZYGO, Middlefield, CA, USA). The contact states of the water droplet on the GP-MA were observed by inverted optic microscopy (ECLIPSE MA100N, Nikon, Tokyo, Japan).

The water contact angle (CA) and slide angle (SA) were measured on a droplet shape analysis (OCA25, Dataphysics, Hamburg, Germany). The volume of the water droplet for the CA and SA measurements was 4 and 8 μL, respectively. The CA and SA were measured at least five times, and the mean values were calculated.

The liquid-solid friction force was tested by the MPCP technique, as established in our previous work [20]. Before the measurement, the GP-MA was mounted on the motor stage and brought into contact with a water droplet of 6 μL. The droplet remained adhered to the capillary with a diameter of 0.3 mm throughout the measuring process. The droplet was driven at a constant speed of 0.3 mm/s relative to the steady capillary, and the displacement of the capillary (*D*) was monitored and recorded simultaneously. The liquid-solid friction force (*F*) of the droplet on the surface can be described as

*F* = *kD*,

where *k* is the spring constant of the capillary. The friction force on one sample was tested no less than five times, and the mean value was calculated.

The temperature control was achieved with a 365 nm UV light source (XC-102, IGEtec., China) with an irradiation area of 20 × 20 mm^2^. The infrared images and temperature data of the samples were acquired by an infrared thermal imaging camera (TiX640 60Hz, Fluke, Everett, WA, USA). A constant irradiation was applied to ensure a stable temperature of the GP-MA during the measurements. The mass of the droplet was monitored by an electronic scale (ME204/02, Mettler-Toledo, Greifensee, Switzerland).

## 3. Results and Discussion

### 3.1. Geometry of the GP-MA

**Figure 1:**
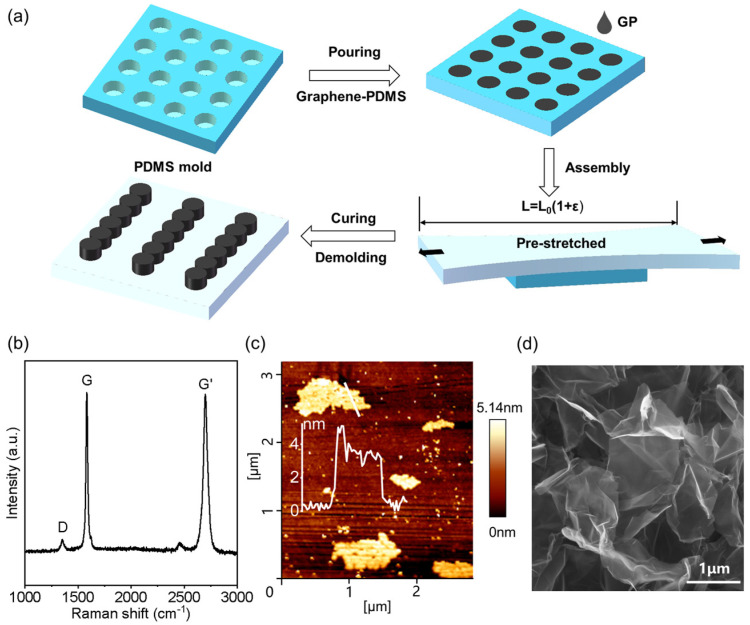
Fabrication of the graphene-PDMS (GP) micropillar array (GP-MA) and the morphologies of the graphene sheets. (**a**) Schematic illustration of the fabrication process; (**b**) Raman spectrum, (**c**) AFM, and (**d**) SEM of the graphene sheets. The inset in (**c**) shows the thickness profile of the graphene sheet.

**Figure 2:**
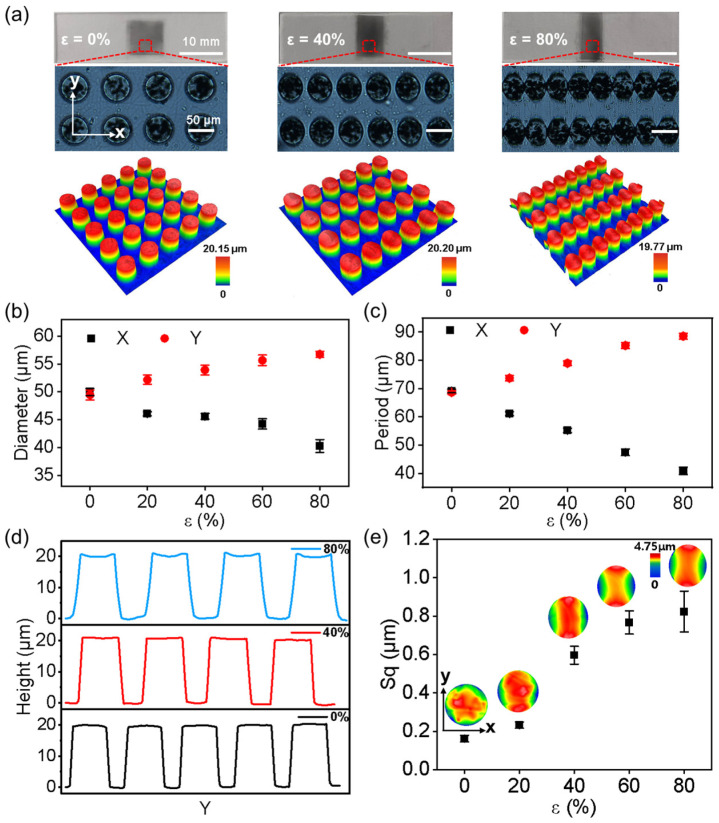
Geometry of the GP-MA. (**a**) The optical image and 3D morphology of the GP-MA with elongations of 0, 40%, and 80%; (**b**) diameter and (**c**) period of the GP-MA along the x and y directions under various elongations; (**d**) typical profile of the GP-MA along the y direction with elongations of 0, 40%, and 80%; (**e**) the root mean square roughness (Sq) of the micropillar top under various elongations. The inset in (**e**) shows the 3D morphology of the micropillar top under various elongations. Each data point in (**b**,**c**,**e**) represents the mean value of at least five measurements. Standard deviations are indicated by error bars.

The GP-MA, which is composed of a PDMS backing layer and GP micropillars, was successfully prepared following the procedure adopted from our previous work (Figure 1a) [18]. Graphene was added in order to offer GP-MA with a photothermal effect, making use of the strong capability of graphene to absorb light with wavelengths across the entire spectrum. The Raman spectrum confirmed the state of graphene rather than graphite (Figure 1b). The G peak at ~1581 cm^−1^ represented the *E_2g_* phonon at the Brillouin zone center, and the G’ peak at ~2698 cm^−1^ originated from the double resonance Raman process in *sp*^2^ carbon. The D peak at ~1352 cm^−1^ gave evidence of the presence of defects, while the intensity ratio between the D and G peaks (~0.15) indicated a small number of defects in the graphene sheets [32,33]. The AFM characterization showed that the thickness of the graphene sheets was 2~4 nm (Figure 1c), suggesting that the graphene sheets had two to four layers [34]. Meanwhile, the graphene sheets had a lateral size of ~1 μm or less. The small lateral size together with the crumpled state of the graphene sheets (Figure 1d) facilitated the dispersion of the graphene sheets in the PDMS matrix and provided a large strain deformation of GP [35]. A 0.8 wt% concentration of graphene was chosen, as the composite has a similar elastic modulus to that of pure PDMS [36,37], which would allow the GP micropillars to be deformed together with the supporting layer. Moreover, the dark field illumination indicated that graphene sheets solely and homogeneously dispersed in the micropillars without diffusion to the backing layer (Figure 2a).

The resulting GP-MA without pre-elongation (*ε* = 0) faithfully replicated the geometry of the micro-holes in the template, showing 50 μm in diameter, 20 μm in height, and 70 μm in period (Figure 2b,c). Here, the direction of pre-stretching was defined as the x direction, while the orthogonal direction of the pre-stretching was defined as the y direction. To quantitatively characterize the geometry of the resulting GP-MA, the corresponding diameter and periodic distances in the x and y directions are noted as *D_x_*, *D_y_*, *P_x_*, and *P_y_*, respectively. With the increase in the pre-elongation (*ε* > 0), the *D_x_* and *P_x_* decreased gradually due to the following relaxation of the pre-stretching. Meanwhile, the *D_y_* and *P_y_* gradually increased due to the Poisson’s ratio effect (Figure 2b,c). That is, as the *ε* increased, the anisotropy of the GP-MA surface increased. For instance, an *ε* of 40% decreased *D_x_* and *P_x_* to 45.58 ± 0.52 μm and 55.16 ± 0.77 μm, respectively; meanwhile, it increased *D_y_* and *P_y_* to 53.91 ± 0.89 μm and 78.96 ± 0.82 μm, respectively. When the *ε* reached 80%, the micropillars presented a “side by side” state in the x direction, as *D_x_* at 40.25 ± 1.17 μm and *P_x_* at 40.91 ± 1.29 μm were quite close. On the other hand, the *D_y_* and *P_y_* reached 56.73 ± 0.55 μm and 88.54 ± 1.01 μm, respectively.

As the GP micropillars have the same elastic modulus as the backing layer, the release of the pre-elongation would cause the synchronized deformation of the GP micropillars with the backing layer. Therefore, the GP micropillars showed elliptical shapes with the supporting layer pre-stretched (Figure 2a, such as an *ε* of 40% and 80%). Moreover, the release of pre-elongation squeezed the micropillar top towards the center along the x direction and stretched the center toward the two sides in the y direction (Figure 2d). As a result, the micropillar top was deformed into a saddle shape (Figure 2e). For instance, at *ε* = 80%, the top of GP micropillars presented an arched shape (high in the middle and short on both sides) along the x direction but a curved shape (short in the middle and high on both sides) along the y direction. Along with the deformation of the micropillar top, the roughness of the micropillar top increased from 0.16 ± 0.01 μm to 0.82 ± 0.11 μm when the pre-elongation was increased (Figure 2e). Therefore, with the increase in *ε*, the anisotropy of the GP-MA also increased in the macro- and microscale.

### 3.2. Interfacial Interaction of a Water Droplet on the GP-MA at Room Temperature

**Figure 3:**
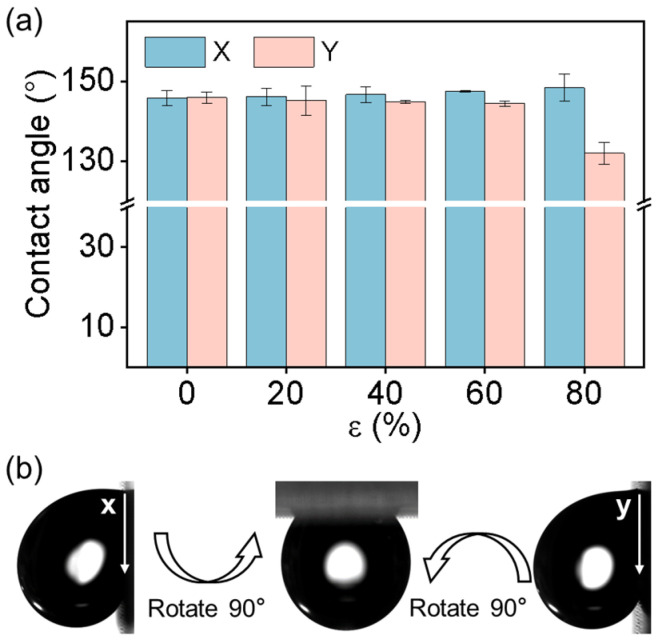
The wettability characterized by the CA and SA measurements. (**a**) The CA of the GP-MA with various elongations; (**b**) typical SA image of the GP-MA. Each data point in (**a**) represents the mean value of at least five measurements. Standard deviations are indicated by error bars.

The wettability of the GP-MA was evaluated by traditional CA and SA measurements. Generally, the wettability of the GP-MA is determined by the surface geometry. When the *ε* was 0, the GP-MA had a CA of 145.8 ± 1.9 and 145.9 ± 1.3° in the x and y directions, respectively (Figure 3a). The negligible difference in the CA in the orthogonal directions suggested that the GP-MA surface was isotropic, which is reasonable, as the period and diameter in the two directions were the same (Figure 2b–d). When the *ε* was increased from 0 to 80%, the CA slightly increased to 148.4 ± 3.4° in the x direction and decreased to 132.0 ± 2.7° in the y direction. The changes in the CA were 2.6° and 13.9°, which meant a difference of 1.8% and 9.5%, respectively. When the backing layer was pre-stretched, i.e., an *ε* up to 60%, the period and the micropillars were anisotropic (Figure 2b,c,e); however, the CAs in the two directions were almost the same. With an *ε* of 80%, the *P_y_* increased to 88.54 ± 1.01 μm, which was quite large compared to the size of the droplet, and a partial penetration of the droplet into the array occurred, resulting in a slight decrease in the CA. Thus, the difference in the CA in the two directions reached 16.4°, which meant a difference of 11.0%. On the other hand, it has been reported that on superhydrophobic surfaces, the uncertainty of one pixel at the diffuse edge and baseline could introduce substantial systematic errors in the CA from 1° to more than 10° [38]. Similarly, the SAs in two directions also showed no difference in that the water droplet did not fall even when the sample was turned upside-down in either the x or y direction (Figure 3b) [18]. That is, the CA and SA measurements could not distinguish the difference between the GP-MA surfaces with various elongations and could not reveal the anisotropy of the GP-MA surfaces.

**Figure 4:**
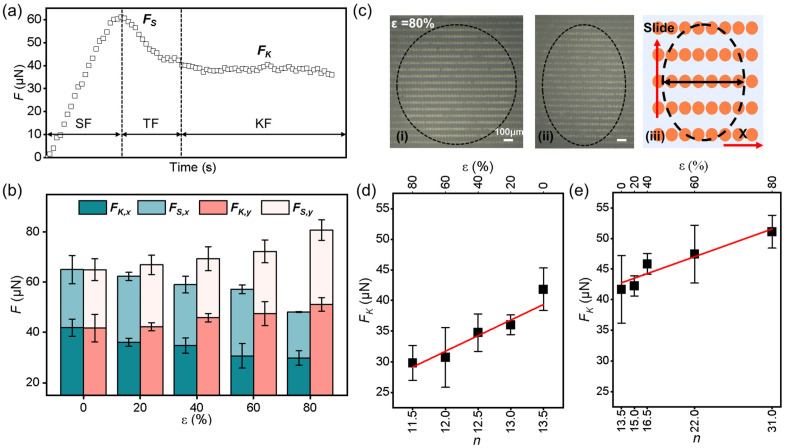
The liquid-solid friction measurement on the GP-MA at room temperature. (**a**) Typical curve showing the region of the static friction (SF), the kinetic friction (KF), and the transition zone of friction (TF); (**b**) the static friction force (*F_S_*) and kinetic friction force (*F_K_*) along two directions on the GP-MA under various elongations; (**c**) the contact interface of the droplet at the initial (i) and KF (ii) states and the corresponding schematic illustration (iii) on the GP-MA with an elongation of 80%; the black dashed lines represent the perimeter of the contact area; the black line at (iii) indicates the short axes of the contact area, where the number of micropillars were counted; (**d**,**e**) influence of the micropillar number on the *F_K_* along the direction of (**d**) the x and (**e**) y under various elongations. Each data point in (**b**,**d**,**e**) represents the mean value of at least five measurements. Standard deviations are indicated by error bars.

The liquid-solid friction of a water droplet on the GP-MA was then determined to examine the surface. Similar to the solid-solid friction, a liquid-solid friction curve also has three sections: static friction, kinetic friction, and the transition zone from static friction to kinetic friction (Figure 4a) [39]. The peak value during the static friction period is considered as the static friction force (*F_S_*), and the mean value during the kinetic friction is calculated to be the kinetic friction force (*F_K_*) [19]. As with the solid-solid friction, *F_S_* is larger than *F_K_*. The friction forces along the x and y directions are then noted as *F_S,x_*, *F_K,x_*, *F_S,y_*, and *F_K,y_*, respectively, for convenience.

When the *ε* was increased from 0 to 80%, the *F_K,x_* decreased from 41.82 ± 3.48 to 29.80 ± 2.8 μN, with a decrease of 28.7%, while the *F_K,y_* increased from 41.66 ± 5.53 to 51.10 ± 2.68 μN, showing an increase of 22.6% (Figure 4b). Compared with the CA measurements when the *ε* increased from 0 to 80%, the differences in the *F_K,x_* and *F_K,y_* were 15.9 and 2.5 times larger, respectively. When the elongation reached 80%, the differences of the *F_K_* (*F_S_*) in two directions reached 71.5% (67.9%), which was more than six times the difference in the CA. That is, the friction measurement can clearly reveal the anisotropy of the surface.

The contact geometry is responsible for the anisotropy friction. At the initial stage, the contact area was a circle (Figure 4c-i), and it changed to an ellipse along the moving direction of the droplet (Figure 4c-ii). Since the friction force is proportional to width of the contact area (short axis of the ellipse), the mean number of micropillars along the short axis (*n*) were then counted (Figure 4c-iii). A larger *n* means a longer pinning front of the droplet during the lateral movement and thus a larger *F_K_*. When the droplet slid in the x direction, the *F_K,x_* decreased from 41.82 ± 3.48 to 29.80 ± 2.8 μN when the *ε* increased from 0 to 80%, showing a linear dependence on *n* (decreased from 13.5 to 11.5, Figure 4d). Similarly, the *F_K,y_* also showed a linear dependence on *n* (increased from 13.5 to 31.0, Figure 4e), while the dependence on *n* was much weaker than for *F_K,x_*. In other words, the regulation of the micropillar arrangement by stress is much more efficient along the x direction. While we could not directly observe the three phase contact line on each micropillar top due to the limitation of our device, we propose an easier movement of the droplet along the x direction than along y direction because of the saddle-shaped micropillar top. That is, along with the period of GP micropillars, the anisotropic micropillar top could also contribute to the anisotropic friction. Once again, the liquid-solid friction strongly suggests the anisotropy of the surface.

### 3.3. Liquid-Solid Friction Measurement on the GP-MA at Elevated Temperatures

**Figure 5:**
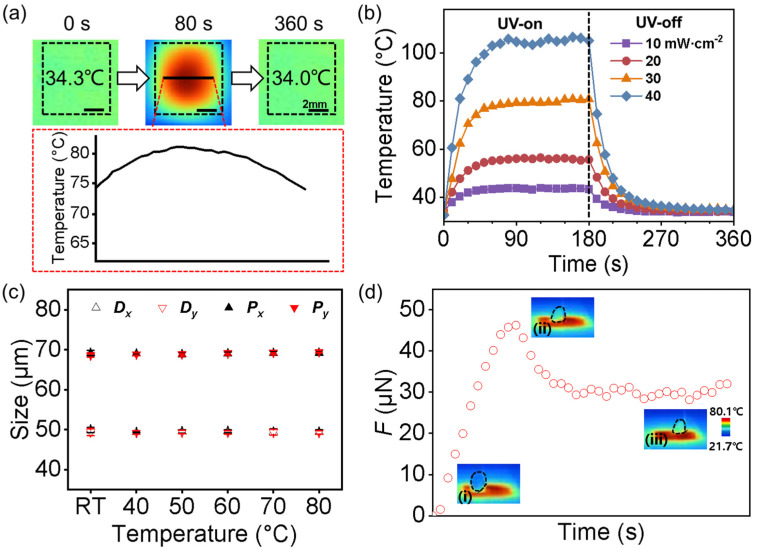
The photothermal effect in the GP-MA. (**a**) Typical infrared image of the GP-MA under UV irradiation at different time. The black line is a typical temperature profile across the GP-MA; (**b**) temperature change of the GP-MA under various UV-light intensities; (**c**) the structure parameters (*D_x_*, *D_y_*, *P_x_*, and *P_y_*) of the GP-MA with an *ε* of 0 at various temperatures; (**d**) typical measuring curve for the friction and infrared images of a water droplet at the initial (i), SF (ii) and KF (iii) states on the GP-MA at 80 °C. Each data point in (**c**) represents the mean value of at least five measurements. Standard deviations are indicated by error bars.

Due to the ability of graphene to absorb light and its photothermal effect [40,41], the temperature of the GP-MA surface could be effectively heated up by UV irradiation remotely. For instance, under a UV irradiation of 30 mW/cm^2^, the GP-MA surface achieved a homogeneous temperature of ~80 °C after 80 s (Figure 5a). By changing the light intensity between 10 and 40 mW/cm^2^, the temperature between 43.9 and 106.5 °C was easily realized (Figure 5b). Stable temperatures ranging from 40 to 80 °C could then be easily and remotely regulated by controlling the light intensity for the following investigation. As the graphene sheets were dispersed solely in the GP micropillars, the temperature of the GP-MA increased while the backing layer remained at room temperature. As a result, the diameter and period of the GP-MA remained the same (*D_x_* = 49.42 ± 0.54 μm, *D_y_* = 49.31 ± 0.42 μm, *P_x_* = 69.17 ± 0.53 μm, and *P_y_* = 69.49 ± 0.31 μm) when the surface temperature was heated up to 80 °C, which was beneficial for the following tests (Figure 5c). When the droplet slid on the heated GP-MA, the bottom part of the droplet would thus be heated up, causing a Marangoni flow inside the droplet (Figure 5d).

**Figure 6:**
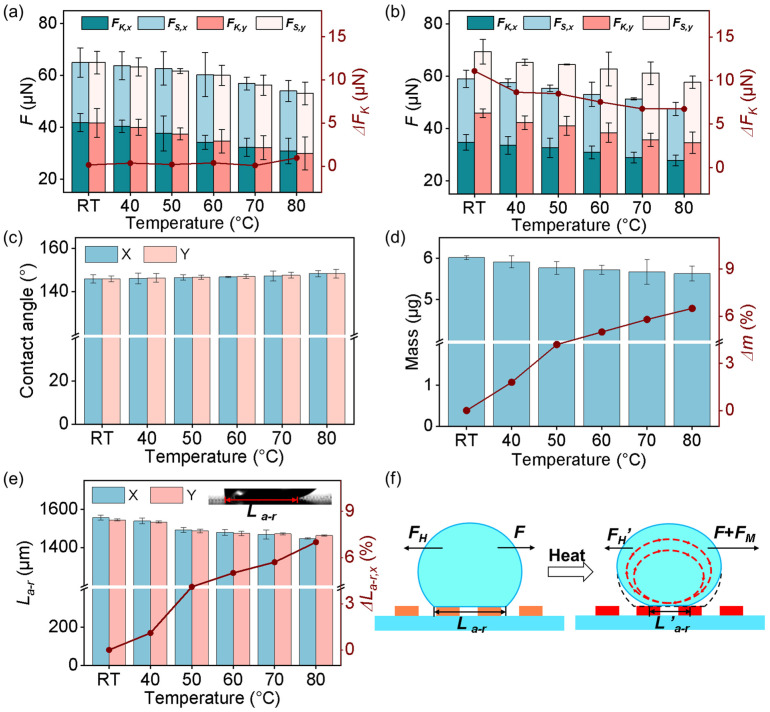
The liquid-solid friction measurement on the GP-MA at elevated temperatures. (**a**,**b**) The friction forces along the x and y directions and the corresponding Δ*F_K_* on the GP-MA with an *ε* of (**a**) 0 and (**b**) 40% at various temperatures; (**c**) the CA along the x and y directions on the GP-MA with an *ε* of 0 at various temperatures; (**d**) mass, the proportion of the mass decrease (Δ*m*) and (**e**) length between the advancing and receding fronts (*L_a__–r_*) and the changing proportion of *L_a__–r_* in the x direction (Δ*L_a__–r,x_*) of a droplet during the friction measurements on the GP-MA with an *ε* of 0 at various temperatures; (**f**) the proposed mechanism for the decrease in the friction force at increased temperature. Each data point in (**a**–**e**) represents the mean value of at least five measurements. Standard deviations are indicated by error bars.

When the temperature of the GP-MA was increased, both the *F_S_* and *F_K_* decreased in both directions. For the sample with *ε* = 0, *F_K,x_* and *F_K,y_* decreased to 30.90 ± 4.9 and 29.94 ± 6.4 μN, with a decrease of 26.1% and 28.1%, respectively, when the temperature increased from room temperature (RT) to 80 °C (Figure 6a). Considering the statistics, there was no difference in the orthogonal directions, showing an anisotropy (Δ*F_K_* = $\left|F_{K,x}-F_{K,y}\right|$) close to 0. The same dependence of friction forces on the temperature was also demonstrated on the GP-MA with an *ε* of 40% (Figure 6b). Different from the GP-MA with *ε* = 0, there were clear differences in the *F_S_* and *F_K_* along the orthogonal directions at all the temperatures tested. Moreover, Δ*F_K_* decreased from 11.09 to 6.74 μN as the temperature increased from RT to 80 °C. That is, the anisotropy changed following the surface temperature.

To understand the temperature dependence of the friction and anisotropy, considering the stability of the structure (Figure 5c), the contact angle (Figure 6c), the mass change (Figure 6d), and the length between the advancing and receding fronts (*L_a__–r_*) during friction (Figure 6e) were investigated. At temperatures ranging from RT to 80 °C, the CA of the GP-MA with an *ε* of 0 ranged from 148.4 ± 2.0° to 148.3 ± 1.4° in both directions (Figure 6c). As a difference of 1.7% in the CA was detected at various temperatures, the influence of temperature on the Young’s equation was thus considered to be negligible. After friction measurement at elevated temperatures, which normally took around 13 s, the evaporation of water would reduce the mass of the droplet. At 80 °C, which is quite close to the boiling point of water, the evaporation was quite fast. As a result, a mass decrease (Δ*m*) of 6.48% in the droplet was detected (Figure 6d). Considering the friction here also follows Amontons’ law that the friction force is proportional to the normal force (here, the droplet weight, *μg*) with a constant friction coefficient of *μ:*

*F* = *μmg*,

a decrease of 6.48%, rather than ~27%, would be expected in the *F_K_*. At lower temperatures, the evaporation of water was much slower, and the Δ*m* was much lower, which meant an even smaller influence on the friction. This strongly suggests there could be other mechanisms contributing to the reduction in the friction force at high temperatures. The *L_a__–r_* during the kinetic friction was also monitored. At RT, an *L_a__–r_* of 1556.38 ± 12.13 μm was detected in the x direction, which decreased by 7.05% (Δ*L_a__–r,x_*) to 1446.86 ± 3.06 μm as the temperature increased to ~80 °C (Figure 6e). The decrease in the *L_a__–r_* means the decrease in the contact area, which determined the contact point between the droplet and the surface and led to the decrease in the friction.

At room temperature, due to Newton’s third law, the tested liquid–solid friction is expressed by the hydrodynamic resistance (*F_H_*):

*F* = *F_H_*,

which includes viscous forces in the droplet and the contact area of the droplet on surfaces [42]. As the contact area decreased at an elevated temperature, the *F_H_* decreased (*F_H_’* < *F_H_*). Additionally, the Δ*T* in the droplet caused spatial variation in the surface tension (Figure 5d), adding a Marangoni force (*F_M_*) [43,44], whose direction was the same as that of the flow in the droplet, to the droplet. The force balance can thus be reconsidered as follows:

*F* + *F_M_* = *F_H_’*.

That is, the Marangoni effect also contributes to the friction reduction. To summarize, the decrease in the mass and contact area and the Marangoni effect contribute together to the friction reduction (Figure 6f).

### 3.4. Liquid-Solid Friction on the GP-MA under Various Elongations

**Figure 7:**
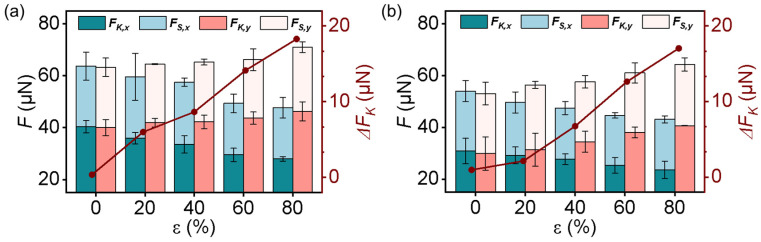
(**a**,**b**) The dependence of the friction forces along the x and y directions and the corresponding Δ*F_K_* at (**a**) 40 °C and (**b**) 80 °C on *ε*. Each data point in (**a**,**b**) represents the mean value of at least five measurements. Standard deviations are indicated by error bars.

At a fixed elevated temperature, the influence of the *ε* on the friction force was then further investigated. At 40 °C, the *F_S,x_* sharply decreased by 25.10% from 63.72 ± 5.41 to 47.73 ± 3.99 μN, and the *F_K,x_* decreased by 30.67% from 40.36 ± 2.36 to 27.98 ± 0.84 μN, correspondingly, when the *ε* increased to 80% (Figure 7a). In contrast, the *F_S,y_* and *F_K,y_* increased from 63.26 ± 3.52 and 40.00 ± 3.09 μN to 70.92 ± 2.04 and 46.26 ± 3.60 μN, with a difference of 10.80% and 15.65%, respectively. As a result, the Δ*F_K_* increased from 0.36 to 18.28 μN, suggesting an increase in the anisotropy with an increase in the *ε.* A similar phenomenon was also found in the GP-MA at 80 °C (Figure 7b). Generally, the friction forces, including *F_S,x_*, *F_S,y_*, *F_K,x_*, and *F_K,y_*, and the anisotropy, were all smaller than that at 40 °C and RT. This confirmed again that the increase in the temperature not only decreased the liquid–solid friction but also decreased the anisotropy.

## 4. Conclusions

Here, we investigated the influence of the temperature on the friction of a water droplet on a GP-MA surface. The GP-MA was composed of GP micropillars supported by a thin layer of pure PDMS. The periods and roughness of the micropillars were regulated by changing the pre-stretching, *ε*, of the PDMS supporting layer. With the increase in the *ε*, the CA and SA showed negligible differences along the orthogonal direction and could not reveal the anisotropy. With an increase in the *ε* (in direction x), the *F_S,y_* and *F_K,y_* increased, while the *F_S,x_* and *F_K,x_* decreased. Meanwhile, the *ε* also increased the anisotropy (Δ*F_K_*). Making use of the photothermal effect of graphene, the temperature of the GP-MA surface could be easily increased. The increase in the temperature decreased the *F_S,x_*, *F_S,y_*, *F_K,x_*, and *F_K,y_*, which originated from the reduced mass and contact area of the droplet and the introduced Marangoni flow in the droplet. Surprisingly, the anisotropy also decreased when the temperature increased, which was the result of the larger structural change in the x direction upon stretching. As the temperature range investigated here is quite common in our daily life, the study allows us to better understand the influence of temperature on wettability, which cannot be well revealed by contact angle measurements. In turn, the investigation here may also pave the way to invent new superwettability materials for high temperatures.

## Data Availability

The data presented in this study are available upon request from the corresponding author.
